# Supplementary material for: Enhancement of Methylene Blue Photodegradation Rate Using Laser Synthesized Ag-Doped ZnO Nanoparticles
Source: Nanomaterials (Basel). 2022 Aug 4;12(15):2677. doi: 10.3390/nano12152677 (PMC9370537; doi:10.3390/nano12152677)
Supplement: Supplementary file 1 [file nanomaterials-12-02677-s001.zip › nanomaterials-1792758-supplementary.pdf]

Supplementary Materials

Enhancement of Methylene Blue Photodegradation Rate Using Laser Synthesized Ag-Doped ZnO Nanoparticles

Damjan Blažeka[1], Rafaela Radičić[1], Dejan Maletić[1], Sanja Živković[2], Miloš Momčilović[2], Nikša Krstulović[1]*

[1] Institute of Physics, Bijenička cesta 46, 10000 Zagreb, Croatia; dblazeka@ifs.hr (D.B.); dmaletic@ifs.hr (D.M.); rradicic@ifs.hr (R.R.); niksak@ifs.hr (N.K.)
[2] VINČA Institute of Nuclear Sciences - National Institute of the Republic of Serbia, University of Belgrade, PO Box 522, 11351 Belgrade, Serbia
sanjaz@vinca.rs (S.Ž.), milos@vin.bg.ac.rs (M.M.)
* Correspondence: niksak@ifs.hr;

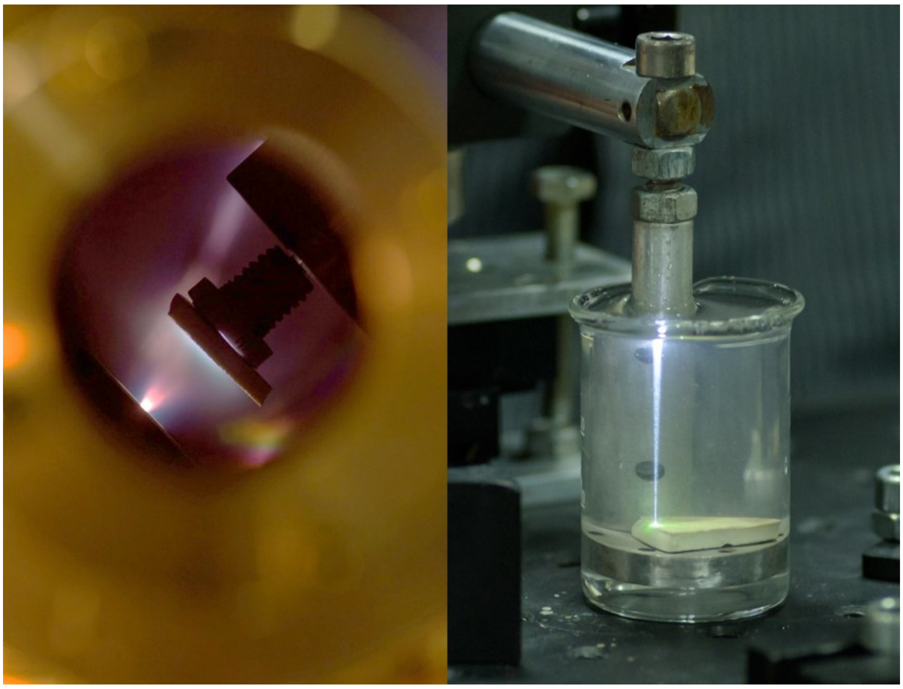

Figure S1. Photographs of experimental setups PLD (on the left) and PLAL (on the right)

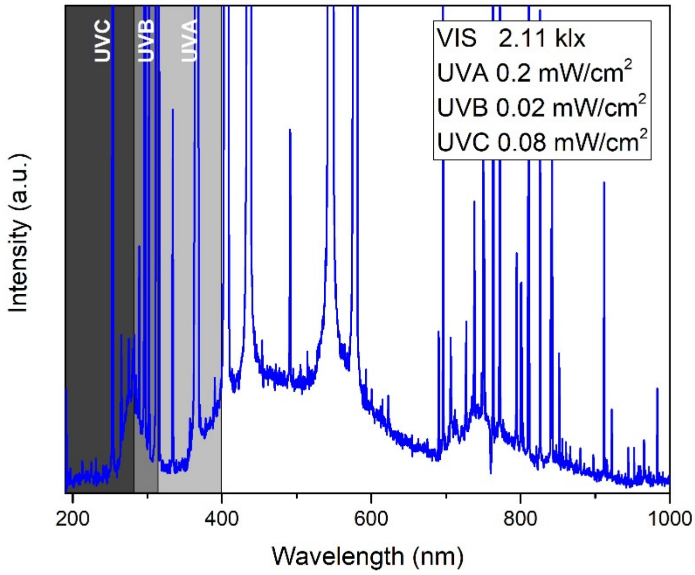

Figure S2. UV lamp emission spectrum with VIS, UVA, UVB and UVC irradiation intensities
